# Conventional Versus Minimally Invasive Mitral Valve Replacement: A Retrospective Comparison of Sternotomy, Mini-Thoracotomy, and Periareolar Approaches

**DOI:** 10.3390/jcm15187200

**Published:** 2026-09-16

**Authors:** Alper Selim Kocaoğlu, Beyza Ece Eral, Cengiz Ovalı

**Affiliations:** 1 Department of Cardiovascular Surgery, Health Sciences University Eskişehir City Health Practice and Research Hospital, 26080 Eskişehir, Turkey; 2 Department of Cardiovascular Surgery, Faculty of Medicine, Eskişehir Osmangazi University, 26040 Eskişehir, Turkey

**Keywords:** mitral valve, heart valve prosthesis implantation, minimally invasive surgical procedures, sternotomy, thoracotomy

## Abstract

**Background/Objectives:** Minimally invasive mitral valve replacement (MVR) is increasingly used as an alternative to median sternotomy, but comparisons of non-robotic, direct-visualization approaches (mini-thoracotomy and periareolar incision) with sternotomy remain sparse, and head-to-head comparisons between the two minimally invasive techniques are even more limited. This study compared early and 1-year outcomes of sternotomy with two direct-visualization minimally invasive approaches for MVR, and compared the two minimally invasive techniques with each other. **Methods:** In this retrospective, single-center study, 196 patients undergoing isolated MVR (tricuspid annuloplasty permitted as a concomitant procedure) between January 2018 and May 2025 were divided into a sternotomy group (*n* = 102) and a minimally invasive group (*n* = 94; periareolar, *n* = 39; mini-thoracotomy, *n* = 55). Perioperative outcomes, pain, cosmetic satisfaction, and 12-month echocardiographic follow-up were compared, with multivariable linear regression used to adjust for confounders. **Results:** Operative and bypass times were longer with the minimally invasive approach, but cross-clamp time was shorter (60.5 ± 13.6 vs. 67.8 ± 8.6 min, *p* < 0.001). The minimally invasive group had less drainage, fewer transfusions, and shorter intensive care unit (ICU) and hospital stays, with higher 30-day cosmetic satisfaction (all *p* < 0.001), confirmed on multivariable analysis. Pain was higher on day 1 but lower by day 7 with the minimally invasive approach (both *p* < 0.001). Complications, mortality, and 1-year valve function were similar between groups. Within the minimally invasive cohort, periareolar access gave higher cosmetic satisfaction, lower pain, and shorter ICU stay and cross-clamp time than mini-thoracotomy (*p* < 0.01 to *p* < 0.001). **Conclusions:** Direct-visualization minimally invasive MVR, performed without robotic assistance, was associated with less bleeding, fewer transfused blood units, and shorter ICU/hospital stay, together with greater cosmetic satisfaction, versus sternotomy; no statistically significant differences were observed in 1-year echocardiographic valve function between groups. Periareolar access may offer additional pain and cosmetic benefits over mini-thoracotomy in appropriately selected patients.

## 1. Introduction

Mitral valve replacement (MVR) remains an important treatment option for patients with advanced mitral valve disease not amenable to repair; treatment strategy more broadly depends on the underlying pathology, valve reparability, surgical risk, anatomy, patient age, prosthesis considerations, and, increasingly, the availability of transcatheter therapies. When replacement is required, it is associated with low mortality and favorable long-term results. Median sternotomy allows wide surgical exposure, but it also carries greater surgical trauma, more postoperative pain, a longer recovery period, and poorer cosmetic results [1].

Over the last twenty years, minimally invasive techniques for mitral valve surgery—including right mini-thoracotomy, periareolar incision, and ministernotomy—have been developed and are now associated with reduced blood loss, fewer transfusions, shorter stays in the intensive care unit and hospital, and greater patient satisfaction [2,3,4]. Large patient series have also shown that mortality and neurological complication rates with these approaches are comparable to sternotomy [2,3]. It should also be noted that much of this supporting literature, including some of the series cited above [1,3], specifically addresses minimally invasive mitral valve repair rather than replacement; the evidence base for minimally invasive mitral valve replacement—the focus of the present study—remains comparatively smaller and merits separate consideration.

Minimally invasive approaches are increasingly chosen by younger patients who prioritize cosmetic outcome; however, most published work centers on robotic or video-assisted methods. Comparisons of non-robotic mini-thoracotomy and periareolar access with sternotomy remain sparse [5], and direct comparisons between the two non-robotic techniques themselves are even more limited.

The present study retrospectively compares early and 1-year outcomes of conventional sternotomy with those of two direct-visualization minimally invasive approaches (mini-thoracotomy and periareolar incision) among patients undergoing isolated mitral valve replacement, with concomitant tricuspid annuloplasty as the only permitted additional procedure. A secondary aim was to compare outcomes between the two minimally invasive access techniques themselves.

## 2. Materials and Methods

This retrospective, single-center study was conducted with the approval of the Ethics Committee for Non-Interventional Clinical Research at Eskişehir Osmangazi University Faculty of Medicine (7 April 2026; Decision No.: 01; E-25403353-050.04-260063186). The study was conducted in accordance with the principles of the Declaration of Helsinki (as revised in Edinburgh, 2000). Because of the retrospective, non-interventional design, the Ethics Committee waived the requirement for individual informed consent.

We reviewed records of patients treated with mitral valve replacement between January 2018 and May 2025 for isolated mitral disease or mitral disease combined with tricuspid involvement. Individuals who additionally required aortic valve surgery, coronary bypass, redo valve surgery, or emergency intervention were not included, leaving a final cohort of 196 eligible patients for analysis. Because mitral valve repair represents a clinically distinct population, patients undergoing repair rather than replacement were excluded from the outset. Among patients with mitral regurgitation, the underlying pathology was mainly rheumatic, with diffuse leaflet thickening/calcification and subvalvular involvement generally considered unsuitable for durable repair; replacement was therefore preferentially performed in these patients.

According to the surgical technique used, patients were classified into a conventional median sternotomy group (*n* = 102) and a minimally invasive group (*n* = 94); the latter was further split into a periareolar-incision subgroup (*n* = 39) and a right mini-thoracotomy subgroup (*n* = 55).

Postoperative pain was assessed using the standard 0–10 Visual Analogue Scale (VAS), recorded prospectively by the ward nursing staff at fixed postoperative timepoints (day 1 and day 7) as part of routine perioperative documentation. Postoperative analgesic management followed a standardized institutional protocol that did not differ between the sternotomy and minimally invasive groups. Cosmetic satisfaction was scored prospectively at the routine 30-day outpatient visit using a 0–10 self-reported satisfaction scale administered by the same nursing staff; because assessment was unblinded, these endpoints remain susceptible to expectation and ascertainment bias (see Section 5).

### 2.1. Surgical Technique

All patients in the conventional group underwent standard median sternotomy. Central aortic and bicaval cannulation was used to institute cardiopulmonary bypass, after which mitral valve replacement was carried out following standard surgical technique.

Before minimally invasive surgery, all patients underwent thoracoabdominal computed tomography (CT) angiography to evaluate peripheral vascular anatomy, and the surgical approach selected reflected both this anatomy and the operating surgeon’s experience. The periareolar technique used a curved incision along the lower border of the areola, whereas the mini-thoracotomy technique used a 4–6 cm anterolateral incision at the inframammary fold; both routes entered the chest through the fourth intercostal space. An Alexis wound protector–retractor (Applied Medical; Rancho Santa Margarita, CA, USA) was placed as the initial retraction device in every case, with a rigid mini-thoracotomy retractor added when the intercostal space proved too narrow.

**Figure 1:**
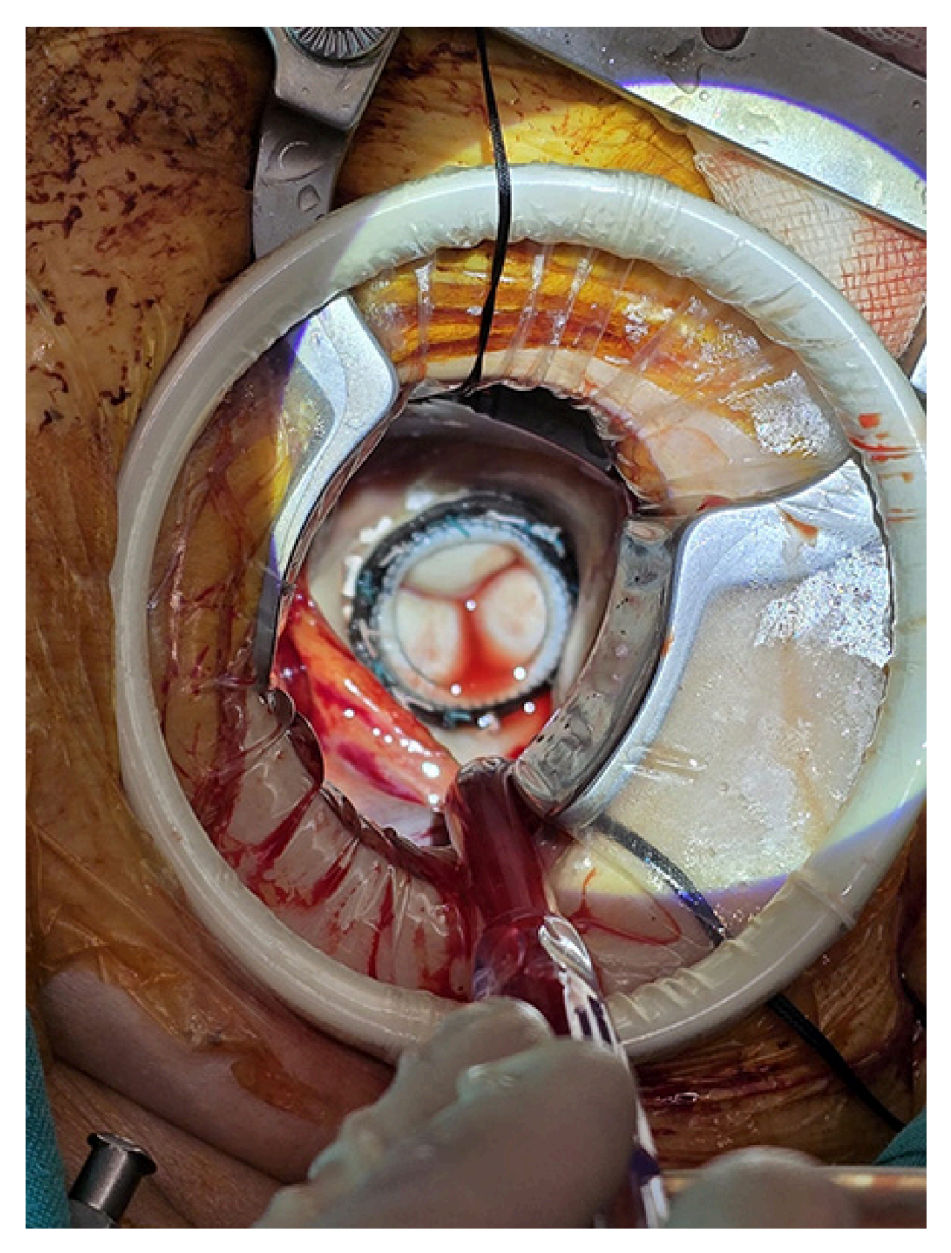
Direct-view image after mitral valve replacement via a minimally invasive approach. This image is published with the patient’s consent.

After careful identification and preservation of the phrenic nerve, the pericardium was incised and retracted with suspension sutures to bring the mitral valve into view (Figure 1). Femoral arteriovenous cannulation was used for cardiopulmonary bypass; once heparin had been administered, the patient was cooled to moderate hypothermia on bypass, and cardiac arrest was induced with antegrade cardioplegia delivered after a transthoracic Chitwood clamp (Scanlan International; Saint Paul, MN, USA) had been applied.

The mitral valve was exposed through a left atriotomy and replaced under direct vision with long-shafted instruments; no patient in this series required thoracoscopic or robotic assistance. Tricuspid annuloplasty was added whenever clinically indicated, after which standard de-airing was performed, bypass was discontinued, and the procedure was completed in the usual manner.

### 2.2. Statistical Methods

Continuous data are expressed as mean ± standard deviation and categorical data as counts with percentages. Patients were grouped by surgical technique—conventional median sternotomy (*n* = 102) versus minimally invasive surgery (*n* = 94)—and the minimally invasive cohort was further separated into periareolar (*n* = 39) and mini-thoracotomy (*n* = 55) subgroups for analysis.

Because several continuous variables departed from a normal distribution, between-group comparisons were carried out with the Wilcoxon rank-sum test. Categorical data were assessed using Pearson’s chi-square test, switching to Fisher’s exact test whenever an expected cell count fell below 5 in a 2 × 2 table.

Independent associations between surgical approach (minimally invasive versus sternotomy as reference) and the key postoperative outcomes—drainage volume, number of transfused blood units, intensive care unit (ICU) stay, and hospital stay—as well as pain and cosmetic endpoints (day 1 and day 7 pain scores, 30-day cosmetic satisfaction) were examined with multivariable linear regression to control for confounding. Each model adjusted for age, sex, body mass index (BMI) > 30 kg/m^2^, diabetes, hypertension, coronary artery disease, chronic obstructive pulmonary disease (COPD)/asthma, baseline left ventricular ejection fraction (LVEF), surgery type, and valve disease type, with findings reported as adjusted β coefficients and 95% confidence intervals (CI). Linear regression was used for all outcomes for consistency and interpretability across endpoints; we acknowledge that count outcomes (number of transfused blood units), right-skewed length-of-stay variables, and bounded pain/cosmetic scores may not fully satisfy the assumptions of ordinary linear regression, and formal model diagnostics were not performed (see Section 5). No formal adjustment for multiple comparisons was applied; in particular, the periareolar-versus-mini-thoracotomy subgroup comparisons in Section 3.5 should be interpreted as exploratory and hypothesis-generating rather than confirmatory.

Box-and-whisker plots were additionally used to visualize how pain scores and cosmetic satisfaction evolved across the study groups.

Statistical significance was defined as a two-sided *p*-value below 0.05. All analyses were conducted in R (version 4.5.3; R Foundation for Statistical Computing, Vienna, Austria).

## 3. Results

The final study population comprised 196 patients: 102 who had mitral valve replacement through conventional median sternotomy and 94 who had the procedure performed via a minimally invasive approach (periareolar incision, *n* = 39; mini-thoracotomy, *n* = 55).

### 3.1. Demographic and Preoperative Characteristics

The two groups were comparable with respect to age (61.3 ± 11.2 vs. 59.5 ± 12.8 years, *p* = 0.298), sex distribution (*p* = 0.10), and baseline left ventricular ejection fraction (56.9 ± 8.6% vs. 57.9 ± 7.6%, *p* = 0.500). Rates of prior infective endocarditis, acute rheumatic fever, diabetes mellitus, hypertension, hyperlipidemia, coronary artery disease, peripheral artery disease, chronic kidney disease, smoking history, and COPD/asthma did not differ meaningfully between groups (all *p* > 0.05) (Table 1).

Valve disease type was likewise distributed similarly between the two groups (Table 1). Rates of isolated mitral regurgitation and of tricuspid regurgitation accompanying mitral stenosis were nearly identical, and although isolated mitral stenosis and tricuspid regurgitation accompanying mitral regurgitation showed some numerical variation between groups, this did not reach statistical significance (*p* = 0.506); these figures are therefore reported descriptively rather than as a formal comparison.

### 3.2. Intraoperative and Early Postoperative Outcomes

Both operative time (137.1 ± 24.0 vs. 125.1 ± 14.8 min, *p* < 0.001) and cardiopulmonary bypass time (88.3 ± 10.6 vs. 84.9 ± 11.3 min, *p* = 0.022) were longer in the minimally invasive group, whereas aortic cross-clamp time was significantly shorter in this group (60.5 ± 13.6 vs. 67.8 ± 8.6 min, *p* < 0.001).

Patients treated minimally invasively had less postoperative drainage (384.9 ± 130.4 vs. 652.2 ± 259.1 mL, *p* < 0.001), received fewer transfused blood units (0.5 ± 0.8 vs. 1.9 ± 1.7 units, *p* < 0.001), and spent less time in the ICU (1.7 ± 2.0 vs. 2.8 ± 2.2 days, *p* < 0.001; equivalent to approximately 40.8 ± 48.0 vs. 67.2 ± 52.8 h) and in the hospital overall (6.0 ± 2.4 vs. 9.0 ± 3.6 days, *p* < 0.001). Mechanical ventilation duration was also shorter in this group (404.5 ± 92.0 vs. 410.8 ± 148.8 min, *p* = 0.042), a statistically significant but clinically small difference of approximately six minutes.

Rates of reoperation for bleeding, wound complications, ICU readmission, pleural effusion requiring intervention, stroke, transient ischemic attack, acute kidney injury, need for permanent dialysis, new-onset atrial fibrillation, and atelectasis were statistically similar between the two groups (all *p* > 0.05), and no patient in either group sustained a postoperative myocardial infarction. In-hospital mortality also did not differ significantly (5.9% vs. 3.2%, *p* = 0.3). Two patients in the minimally invasive group required permanent pacemaker implantation (Table 2), and three femoral cannulation-related vascular complications occurred in this group—two embolectomies and one vascular repair.

### 3.3. Echocardiographic Follow-Up

Among patients who survived to hospital discharge, 12-month echocardiographic follow-up was available for all patients (96 in the sternotomy group and 91 in the minimally invasive group). By the 12-month visit, no statistically significant between-group difference was observed in ejection fraction (55.2 ± 5.4% vs. 57.8 ± 6.9%, *p* = 0.075); mitral prosthetic valve function was well preserved in both groups, and neither severe prosthetic stenosis nor regurgitation was identified in any patient. Tricuspid valve function was also similar across groups (Supplementary Table S1). Because this comparison was restricted to hospital survivors and no formal equivalence or non-inferiority testing was performed, these findings should be interpreted as an absence of detected differences in the measured parameters rather than as formal evidence of equivalence.

### 3.4. Pain and Cosmetic Outcomes

**Figure 2:**
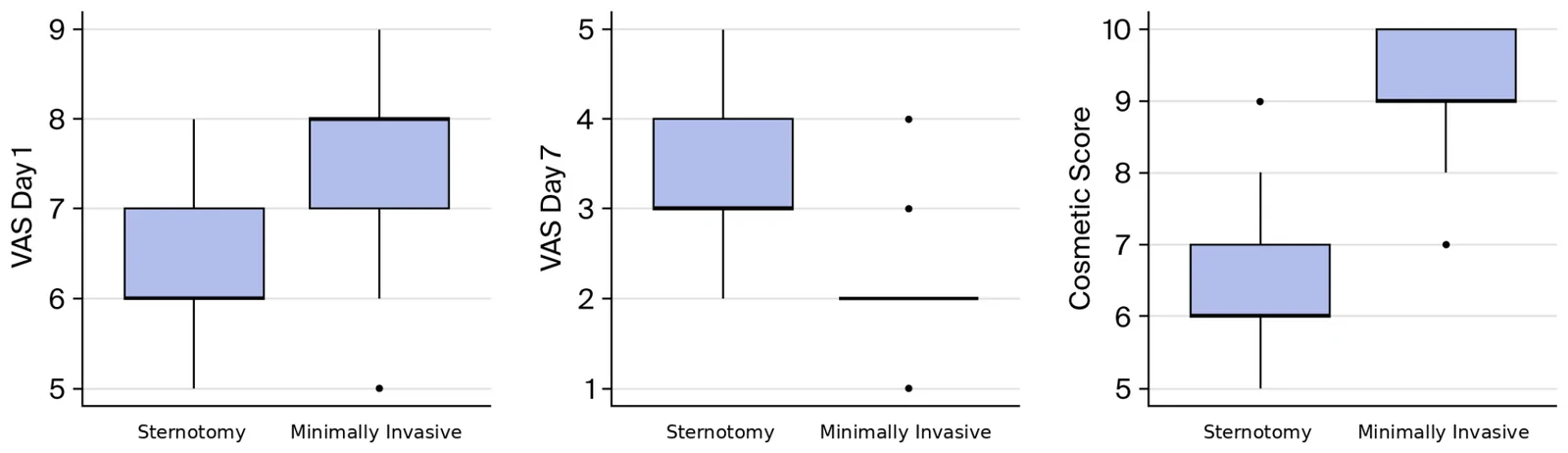
Comparison of postoperative pain scores and cosmetic satisfaction between the conventional sternotomy and minimally invasive groups. Box plots show the median and interquartile range (IQR); whiskers extend to the most extreme values within 1.5 × IQR, and dots represent outliers beyond this range.

**Figure 3:**
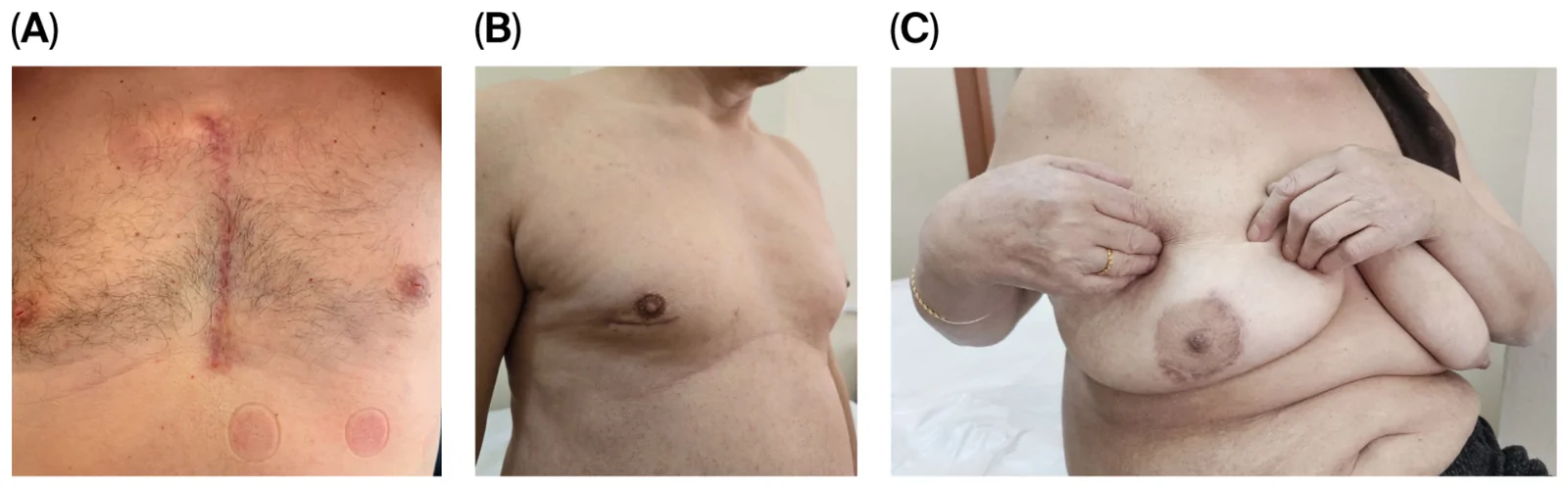
Representative postoperative scars: (**A**) median sternotomy, (**B**) right mini-thoracotomy, and (**C**) periareolar incision. These images are published with the patients’ consent.

Cosmetic satisfaction at 30 days was higher in the minimally invasive group (9.2 ± 0.8 vs. 6.3 ± 0.7, *p* < 0.001). Pain scores showed the opposite pattern over time: on postoperative day 1 they were higher in the minimally invasive group (7.4 ± 0.9 vs. 6.4 ± 0.7, *p* < 0.001), but by day 7 they had fallen below those of the sternotomy group (2.0 ± 0.7 vs. 3.3 ± 0.5, *p* < 0.001) (Table 2, Figure 2 and Figure 3).

### 3.5. Comparison of the Periareolar and Mini-Thoracotomy Approaches

Within the minimally invasive cohort, in this exploratory, hypothesis-generating comparison (surgical approach was not randomly allocated within the minimally invasive group, and no adjustment for multiplicity or confounding was applied), several outcomes differed significantly between the periareolar and mini-thoracotomy subgroups: cosmetic satisfaction was higher (9.5 ± 0.7 vs. 9.0 ± 0.8, *p* < 0.001), pain scores on postoperative days 1 and 7 were lower (*p* < 0.01), and mechanical ventilation time (*p* = 0.024), ICU stay (*p* < 0.001), and aortic cross-clamp time (*p* = 0.002) were all shorter. Drainage volume, bypass time, operative time, transfusion requirement, and hospital stay did not differ significantly between the two subgroups (Table 3). The Alexis wound-protective retractor served as the primary retraction device in both subgroups; a rigid mini-thoracotomy retractor was added when intercostal space was inadequate, which occurred in 11 of 39 periareolar patients (28.2%) compared with 35 of 55 mini-thoracotomy patients (63.6%).

### 3.6. Multivariable Analysis

After adjustment for the measured confounders, multivariable regression showed that the minimally invasive approach remained independently associated with lower postoperative drainage volume (β = −248.58; 95% CI: −315.44 to −181.72; *p* < 0.001), fewer transfused blood units (β = −1.38; 95% CI: −1.81 to −0.95; *p* < 0.001), shorter ICU stay (β = −1.05; 95% CI: −1.72 to −0.38; *p* = 0.002), and shorter hospital stay (β = −3.09; 95% CI: −4.09 to −2.10; *p* < 0.001). It was likewise independently associated with higher day-1 pain scores (β = 1.33; 95% CI: 1.08 to 1.58; *p* < 0.001), lower day-7 pain scores (β = −1.28; 95% CI: −1.49 to −1.07; *p* < 0.001), and greater cosmetic satisfaction at 30 days (β = 2.91; 95% CI: 2.67 to 3.15; *p* < 0.001) (Table 4). As noted in the Methods and Limitations, these estimates derive from linear regression models that were not individually assessed for distributional fit and do not incorporate propensity-based adjustment; they are therefore best interpreted as adjusted associations rather than causal effect estimates.

## 4. Discussion

A substantial body of literature indicates that minimally invasive mitral valve surgery allows quicker recovery with reduced surgical trauma. Grant et al. [6], in a multicenter propensity-score-matched cohort, reported lower transfusion rates and shorter hospitalization with the minimally invasive approach while mortality and neurological complication rates remained equivalent to sternotomy; comparable conclusions emerged from the series reported by Pojar et al. and Cetinkaya et al. [7,8]. Our data align with these reports, and our multivariable analyses show that the minimally invasive technique remained independently associated with lower drainage, fewer transfused blood units, and shorter intensive care and hospital stay, after adjustment for the measured confounders.

Longer operative and bypass times in minimally invasive surgery are expected, given the added steps required for peripheral cannulation and the constraints of limited exposure. Notably, however, cross-clamp time was significantly shorter in our minimally invasive group—contrary to what most published series describe [9]—which we attribute to a standardized, reproducible operative sequence developed with experience in the technique, rather than to any specific device or instrument used exclusively in the minimally invasive group (for example, suture-fastening devices such as Cor-Knot (LSI Solutions; Victor, NY, USA) were not used differentially between the two groups). We believe this efficiency gain may be reproducible in other centers adopting a similarly standardized approach, once past the initial learning curve.

The cosmetic advantage of the minimally invasive approach is clinically meaningful, particularly for younger and female patients.

The reversal in pain scores over time—higher on day 1 but lower by day 7 in the minimally invasive group—most plausibly reflects the different mechanisms underlying pain in the two approaches. We attribute the early pain increase mainly to transient intercostal stretching during retraction, which produces traction/compression neuropraxia of the intercostal nerve; this mechanism would explain why the pain is sharp, well localized, and neuropathic in character, and why limited or rib-sparing retraction strategies have been reported to lessen it [10]. Sternotomy pain, by contrast, resolves more gradually because it depends on weeks of bone healing—a pathophysiological distinction that plausibly underlies a pain profile that disfavors the minimally invasive approach early on but favors it later. Perhaps the most clinically relevant observation in our series is that the differences in pain, number of transfused blood units, and ICU length of stay remained consistent in both unadjusted comparisons and confounder-adjusted multivariable analyses. In a similar vein, we attribute lower drainage and transfusion volumes to avoidance of the diffuse bone-marrow bleeding surface and extensive soft-tissue dissection inherent to sternotomy, while the shorter ICU stay likely reflects reduced surgical trauma together with earlier extubation and mobilization. Taken together, these observations suggest that the minimally invasive approach is associated with not only better cosmesis but also a faster overall clinical recovery; as a retrospective, non-randomized comparison, however, this should be regarded as an association rather than a proven causal effect.

On echocardiographic follow-up, no significant between-group differences were observed in ejection fraction, prosthetic function, or residual pathology at one year—findings that are consistent with recent meta-analytic data [9,11].

A particular strength of this study is the head-to-head comparison, within a single patient population, of two direct-visualization minimally invasive techniques performed entirely without robotic or thoracoscopic assistance. This distinction matters clinically: it suggests that the principal benefits associated with minimally invasive mitral valve surgery—less bleeding, shorter intensive care unit and hospital stay, and superior cosmesis—are attainable using only long-shafted instruments and direct surgical visualization of the operative field, without the capital cost, dedicated equipment, and steep learning curve that robotic or video-thoracoscopic platforms demand. Periareolar access was associated with more favorable cosmetic satisfaction, pain, ICU-stay, and cross-clamp outcomes than mini-thoracotomy in this exploratory, non-randomized subgroup analysis, with no statistically significant difference in hospital stay, suggesting it may be a safe option when patient anatomy is favorable.

Because both the periareolar and mini-thoracotomy routes enter the chest through the same fourth intercostal space, the two techniques differ chiefly in the location and course of the skin incision—which raises the question of what actually drives the observed differences in pain and ICU duration. One possibility relates to the incision itself: the curved periareolar incision passes through breast tissue without directly dividing the pectoral muscle, whereas the more linear mini-thoracotomy incision transects both the pectoral and intercostal muscle planes; this could lower the pain contribution from the superficial chest-wall muscle and fascia even though the same intercostal space is used. A second possibility concerns retraction: although the Alexis wound-protective retractor was the primary device in both subgroups, a rigid mini-thoracotomy retractor was needed far more often in the mini-thoracotomy group (63.6%) than in the periareolar group (28.2%), and this device’s direct, focused force on the ribs may itself have worsened intercostal nerve compression-traction and thus pain in the mini-thoracotomy group [12]. Selection bias may also have played a role in the ICU-stay difference, since periareolar access may have been chosen preferentially for anatomically favorable patients—typically those with thinner chest walls; the unequal subgroup sizes and the retrospective, non-randomized design temper this interpretation, however. Prospective work using a standardized retraction protocol will be needed to determine which of these mechanisms actually explains the difference.

Robotic mitral valve surgery represents a further step in minimally invasive technique, yet its adoption remains limited by high acquisition costs, a demanding learning curve, and infrastructure requirements. Recent reports describe less bleeding and shorter hospitalization with robotic replacement, at the cost of markedly longer operative and cross-clamp times [13,14,15,16], with proficiency generally reached only after roughly 90 procedures [17]. The direct-visualization techniques described here achieve many of the same benefits without a robotic platform, offering a practical option for centers that lack robotic capability.

Registry data from the Mini-Mitral International Registry and the Society of Thoracic Surgeons (STS) database confirm that, given appropriate patient selection, minimally invasive mitral surgery is safe, effective, and increasingly adopted [18,19,20]; the near-even split between our conventional and minimally invasive groups mirrors this broader trend.

For patients judged high-risk or inoperable for conventional surgery, transcatheter mitral valve implantation has emerged as an additional therapeutic option, and its technical and patient-selection challenges continue to shape its role relative to surgical repair or replacement [21]. Our findings pertain specifically to surgically eligible patients and are intended to inform the choice of surgical access rather than the broader decision between surgical and transcatheter therapy.

## 5. Limitations

Several limitations should be considered. This was a retrospective, single-center study in which surgical approach was chosen according to patient anatomy and surgeon experience rather than randomly allocated; treatment allocation was therefore not random, and residual confounding by indication cannot be excluded despite multivariable adjustment. We did not perform propensity-score matching, inverse probability weighting, or overlap weighting: with 196 patients distributed across three surgical-approach groups—including a periareolar subgroup of only 39 patients—and multiple relevant covariates, we judged that a newly specified propensity model could not be reliably estimated and validated (adequate covariate balance, sufficient common support, and stable weights) in this dataset, and that presenting such an analysis risked conveying false analytic precision rather than genuinely strengthening causal inference. Consequently, the associations reported here—including the multivariable-adjusted estimates—should be interpreted as associative rather than causal, and we have revised the wording throughout the manuscript accordingly. The study period spans 2018–2025, during which surgical experience, perioperative protocols, and the adoption of minimally invasive surgery may have evolved; calendar year/era and surgeon-specific effects were not included as covariates in the adjustment models, and a formal sensitivity analysis for learning-curve or treatment-era effects was not performed. Multivariable analyses used linear regression for all outcomes for consistency and interpretability; count outcomes (number of transfused blood units), right-skewed length-of-stay variables, and bounded pain/cosmetic scores may not fully satisfy the assumptions of ordinary linear regression, and formal model diagnostics were not reported—generalized linear, count, or ordinal regression models may be more appropriate and would help confirm the robustness of these associations in future work. No formal adjustment for multiplicity was applied across the numerous comparisons performed, particularly in the periareolar-versus-mini-thoracotomy analysis; these subgroup comparisons were not randomized and should be regarded as exploratory and hypothesis-generating rather than confirmatory.

We also lacked a robotic comparison arm, limited follow-up to 12 months, had a relatively small periareolar subgroup, and did not assess patient-centered endpoints such as quality of life, return-to-work time, or cost-effectiveness. Pain and cosmetic outcomes were assessed without blinding, which may be particularly relevant given that these endpoints are susceptible to expectation and ascertainment bias. Because only replacement patients were included, our conclusions cannot be extended to populations eligible for valve repair. The subgroup differences in pain and ICU stay in particular warrant cautious interpretation given the modest sample size, lack of randomization, and absence of adjustment for confounding within this comparison; prospective studies with a standardized dissection protocol and propensity-based or randomized allocation would help clarify the underlying mechanism.

## 6. Conclusions

Compared with sternotomy, minimally invasive mitral valve replacement was associated with less bleeding, fewer transfused blood units, shorter ICU and hospital stays, and greater cosmetic satisfaction, despite longer operative and bypass times; postoperative complication rates were similar, and no statistically significant differences were observed in 1-year echocardiographic valve function. Within the minimally invasive group, the periareolar technique was associated with lower pain scores and greater cosmetic satisfaction than mini-thoracotomy, suggesting that it may offer additional benefits in appropriately selected patients. Overall, our findings suggest that favorable outcomes associated with minimally invasive mitral valve replacement can be achieved using direct surgical visualization and standard long-shafted instruments, without reliance on robotic or thoracoscopic assistance. Because this approach avoids the capital investment, dedicated equipment, and specialized training that robotic or video-thoracoscopic platforms require, it may represent a practical and cost-accessible alternative to sternotomy for appropriately selected patients, including in centers with limited resources or without robotic capability.

## Figures and Tables

**Table 1:** Demographic Characteristics of Patients.

Variable	Conventional *n* = 102 ^†^	Minimally Invasive *n* = 94 ^†^	*p*-Value ^‡^
Age (years)	61.3 ± 11.2	59.5 ± 12.8	0.298
Preoperative LVEF (%)	56.9 ± 8.6	57.9 ± 7.6	0.500
**Gender**			0.10
Female	66 (65%)	71 (76%)	
Male	36 (35%)	23 (24%)	
**Comorbidities and Risk Factors**			
Infective endocarditis	5 (4.9%)	0 (0%)	0.060
History of acute rheumatic fever	14 (14%)	12 (13%)	0.8
Diabetes mellitus (DM)	24 (23.5%)	27 (28.7%)	0.408
Hypertension (HT)	51 (50%)	52 (55%)	0.5
Hyperlipidemia (HPL)	20 (19.6%)	23 (24.5%)	0.441
Coronary artery disease (CAD)	17 (17%)	18 (19%)	0.7
Peripheral artery disease (PAD)	2 (2.0%)	6 (6.4%)	0.2
Chronic kidney disease (CKD)	3 (2.9%)	1 (1.1%)	0.6
Smoking	40 (39%)	26 (28%)	0.087
COPD/Asthma	13 (13%)	10 (11%)	0.6
**Surgery Performed**			**0.22**
MVR	40 (39%)	45 (48%)	
MVR + TAP	62 (61%)	49 (52%)	
**Valve Disease Type**			**0.506**
Mitral regurgitation (MR)	15 (14.7%)	14 (15%)	
Mitral stenosis (MS)	25 (24.5%)	31 (33%)	
MR + tricuspid regurgitation (TR)	53 (52%)	44 (47%)	
MS + TR	9 (8.8%)	5 (5.3%)	

^†^ Mean ± SD; *n* (%). ^‡^ Wilcoxon rank-sum test; Pearson’s chi-squared test or Fisher’s exact test, as appropriate. DM, diabetes mellitus; HT, hypertension; HPL, hyperlipidemia; CAD, coronary artery disease; PAD, peripheral artery disease; CKD, chronic kidney disease; COPD, chronic obstructive pulmonary disease; LVEF, left ventricular ejection fraction; MVR, mitral valve replacement; TAP, tricuspid annuloplasty; MR, mitral regurgitation; MS, mitral stenosis; TR, tricuspid regurgitation. Bold row labels denote category/summary headings for the rows immediately below.

**Table 2:** Intraoperative, Postoperative, and Pain/Cosmetic Outcomes.

Variable	Conventional *n* = 102 ^†^	Minimally Invasive *n* = 94 ^†^	*p*-Value ^‡^
Operating time (min)	125.1 ± 14.8	137.1 ± 24.0	<0.001
Cardiopulmonary bypass time (min)	84.9 ± 11.3	88.3 ± 10.6	0.022
Aortic cross-clamp duration (min)	67.8 ± 8.6	60.5 ± 13.6	<0.001
Postoperative mechanical ventilation time (min)	410.8 ± 148.8	404.5 ± 92.0	0.042
Postoperative drainage volume (mL)	652.2 ± 259.1	384.9 ± 130.4	<0.001
Postoperative blood transfusion (units)	1.9 ± 1.7	0.5 ± 0.8	<0.001
Length of stay in the intensive care unit (days)	2.8 ± 2.2	1.7 ± 2.0	<0.001
Postoperative hospital stay (days)	9.0 ± 3.6	6.0 ± 2.4	<0.001
Reoperation due to bleeding	6 (5.9%)	2 (2.1%)	0.2
Surgical wound drainage	9 (8.8%)	5 (5.3%)	0.341
Surgical revision for wound complications	7 (6.9%)	5 (5.3%)	0.770
ICU readmission	3 (2.9%)	1 (1.1%)	0.6
Pleural effusion requiring intervention	4 (3.9%)	2 (2.1%)	0.7
Postoperative stroke	1 (1.0%)	0 (0%)	>0.9
Postoperative transient ischemic attack (TIA)	2 (2.0%)	2 (2.1%)	>0.9
Postoperative myocardial infarction	0	0	NA
Postoperative acute kidney injury (AKI)	6 (6%)	2 (2.1%)	0.282
Postoperative need for permanent dialysis	2 (2.0%)	0 (0%)	0.5
New-onset atrial fibrillation	17 (17%)	15 (16%)	0.9
**Postoperative pacing requirement**			0.2
None	83 (81%)	80 (85%)	
Temporary pacing	19 (19%)	12 (13%)	
Permanent pacemaker implantation	0 (0%)	2 (2.1%)	
Postoperative atelectasis	7 (6.9%)	9 (9.6%)	0.489
In-hospital mortality	6 (5.9%)	3 (3.2%)	0.3
**Pain and Cosmetic Outcomes**			
30-day cosmetic satisfaction score (0–10)	6.3 ± 0.7	9.2 ± 0.8	<0.001
Visual pain score on postoperative day 1 (0–10)	6.4 ± 0.7	7.4 ± 0.9	<0.001
Visual pain score on postoperative day 7 (0–10)	3.3 ± 0.5	2.0 ± 0.7	<0.001

^†^ Mean ± SD; *n* (%). ^‡^ Wilcoxon rank-sum test; Pearson’s chi-squared test or Fisher’s exact test, as appropriate. NA, not applicable. Bold row labels denote category/summary headings for the rows immediately below. Surgical revision for wound complications comprised: thoracotomy incision with vacuum-assisted closure (VAC) and closure (0 vs. 2), sternotomy incision with VAC and closure (5 vs. 0), sternotomy incision tissue flap (2 vs. 0), femoral incision site revision (0 vs. 1), and thoracotomy incision debridement with primary closure (0 vs. 2), in the conventional and minimally invasive groups, respectively.

**Table 3:** Comparison of Postoperative Outcomes in Patients Undergoing Periareolar Incision and Mini-Thoracotomy (Exploratory Subgroup Analysis).

Variable	Periareolar *n* = 39 ^†^	Mini-Thoracotomy *n* = 55 ^†^	*p*-Value ^‡^
30-day cosmetic satisfaction score (0–10)	9.5 ± 0.7	9.0 ± 0.8	<0.001
Visual pain score on postoperative day 1 (0–10)	6.8 ± 0.8	7.9 ± 0.7	<0.001
Visual pain score on postoperative day 7 (0–10)	1.8 ± 0.9	2.2 ± 0.5	0.002
Postoperative mechanical ventilation time (min)	389.8 ± 59.5	425.3 ± 122.2	0.024
Postoperative drainage volume (mL)	366.5 ± 117.6	398.0 ± 138.3	0.4
Postoperative blood transfusion (units)	0.3 ± 0.6	0.6 ± 0.9	0.053
Length of stay in the intensive care unit (days)	1.3 ± 0.4	2.1 ± 0.5	<0.001
Postoperative hospital stay (days)	5.3 ± 1.9	5.8 ± 1.9	0.198
Aortic cross-clamp duration (min)	59.5 ± 11.9	67.0 ± 10.8	0.002
Cardiopulmonary bypass time (min)	83.7 ± 12.2	85.7 ± 10.7	0.3
Operating time (min)	139.0 ± 27.0	134.2 ± 20.5	0.452

^†^ Mean ± SD. ^‡^ Wilcoxon rank-sum test.

**Table 4:** Adjusted Associations Between the Minimally Invasive Approach and Postoperative Outcomes.

Outcome	β (95% CI)	*p*-Value
**Pain and Cosmetic Outcomes**		
Pain score on postoperative day 1	1.33 (1.08 to 1.58)	<0.001
Pain score on postoperative day 7	−1.28 (−1.49 to −1.07)	<0.001
30-day cosmetic satisfaction score	2.91 (2.67 to 3.15)	<0.001
**Clinical Outcomes**		
Postoperative drainage volume (mL)	−248.58 (−315.44 to −181.72)	<0.001
Postoperative blood transfusion (units)	−1.38 (−1.81 to −0.95)	<0.001
Intensive care unit stay (days)	−1.05 (−1.72 to −0.38)	0.002
Postoperative hospital stay (days)	−3.09 (−4.09 to −2.10)	<0.001

β coefficients represent the adjusted mean difference for the minimally invasive approach compared with sternotomy. Models were adjusted for age, sex, body mass index > 30 kg/m^2^, diabetes mellitus, hypertension, coronary artery disease, chronic obstructive pulmonary disease/asthma, preoperative left ventricular ejection fraction, type of surgery, and type of valve disease. CI, confidence interval.

## Data Availability

The data presented in this study are available on reasonable request from the corresponding author.

## Supplementary Materials

The following supporting information can be downloaded at: https://www.mdpi.com/article/10.3390/jcm15187200/s1, Table S1: Postoperative Echocardiographic Follow-up Findings.
