# Supplementary material for: Conventional Versus Minimally Invasive Mitral Valve Replacement: A Retrospective Comparison of Sternotomy, Mini-Thoracotomy, and Periareolar Approaches
Source: J Clin Med. 2026 Sep 16;15(18):7200. doi: 10.3390/jcm15187200 (PMC13607934; doi:10.3390/jcm15187200)
Supplement: Supplementary file 1 [file jcm-15-07200-s001.zip › jcm-4559370-supplementary.pdf]

Supplementary Materials

Conventional versus Minimally Invasive Mitral Valve Replacement: A Retrospective Comparison of Sternotomy, Mini-Thoracotomy, and Periareolar Approaches

Table S1. Postoperative Echocardiographic Follow-up Findings.

Variable	Conventional n = 96†	Minimally Invasive n = 91†	p-value‡
LVEF at 1 month postoperatively	56.3 ± 6.6	57.5 ± 7.3	0.079
LVEF at 12 months postoperatively	55.2 ± 5.4	57.8 ± 6.9	0.075
**Mitral prosthetic valve regurgitation at 12 months**			
None	94 (98%)	90 (98.9%)	
Mild	2 (2%)	1 (1.1%)	
Moderate	0 (0%)	0 (0%)	
Severe	0 (0%)	0 (0%)	
**Mitral prosthetic valve stenosis at 12 months**			
None	95 (98.9%)	90 (98.9%)	
Mild	1 (1.1%)	1 (1.1%)	
Moderate	0 (0%)	0 (0%)	
Severe	0 (0%)	0 (0%)	
**Tricuspid regurgitation at 12 months**			
None	78 (81.2%)	76 (83.6%)	
Mild	17 (17.7%)	14 (15.3%)	
Moderate	1 (1.1%)	1 (1.1%)	
Severe	0 (0%)	0 (0%)	

† Mean ± SD; n (%).

‡ Wilcoxon rank-sum test; Pearson's chi-squared test or Fisher's exact test, as appropriate.

LVEF, left ventricular ejection fraction.
